# Supplementary material for: The oxidized phospholipid oxPAPC protects from septic shock by targeting the non-canonical inflammasome in macrophages
Source: Nat Commun. 2018 Mar 8;9:996. doi: 10.1038/s41467-018-03409-3 (PMC5843631; doi:10.1038/s41467-018-03409-3)
Supplement: Supplementary file 1 — Supplementary Information [file 41467_2018_3409_MOESM1_ESM.pdf]

Supplementary Information

The oxidized phospholipid oxPAPC protects from septic shock by targeting the non-canonical inflammasome in macrophages

Chu et al.

Supplementary Figures

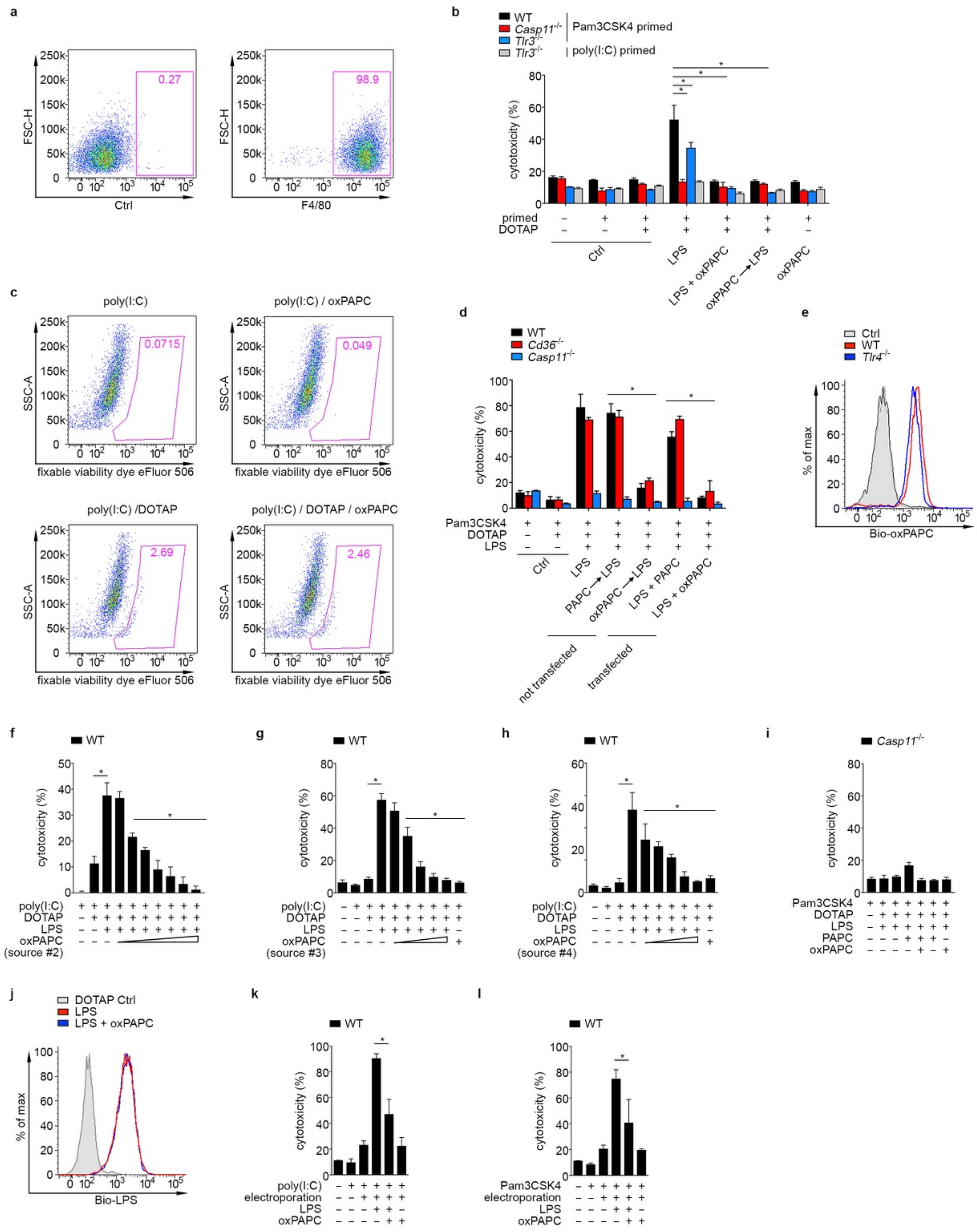

Supplementary Figure 1 | oxPAPC inhibits cytosolic LPS-induced pyroptosis a, BMDMs were analyzed for F4/80 expression by flow cytometry, indicating positive cells (%). **b,** Peritoneal macrophages (PMs) of the indicated genotypes were primed with Pam3CSK4 (1 μg ml^{-1}) or poly(I:C) (1 μg mL^{-1}) for 6h and then transfected with LPS (50 ng per well) and either co-transfected with oxPAPC (100 ng per well) or pre-treated with oxPAPC (100 ng per well, indicated by an arrow) and LDH release after 2h was determined and presented as % cytotoxicity compared to maximum LDH release. **c,** BMDMs were primed with poly(I:C) (1 μg mL^{-1}) for 6h and then either left untreated or were treated with oxPAPC (20 μg mL^{-1}) (top panels) or were mock transfected or were transfected with oxPAPC (1.5 μg per 0.5 x 10^6 cells) (bottom panels) for 30 min and analyzed for cell viability by flow cytometry. **d,** WT, *Casp11*$^{-/-}$ and *Cd36*$^{-/-}$ BMDMs were primed with Pam3CSK4 (1 μg mL^{-1}) for 6h and then transfected with LPS (50 ng per well) and either co-transfected with oxPAPC (100 ng per well) or pre-treated with oxPAPC (100 ng per well, indicated by an arrow) and LDH release after 2h was determined and presented as % cytotoxicity compared to maximum LDH release. **e,** WT and *Tlr4*$^{-/-}$ BMDMs were treated with biotinylated oxPAPC (20 μg mL^{-1}) (Bio-oxPAPC) for 30 min and intracellular oxPAPC quantified by flow cytometry. **f-h,** WT BMDMs were primed with poly(I:C) (1 μg mL^{-1}) for 6h and then transfected with LPS (50 ng per well) and (**f**) oxPAPC (10, 30, 50, 75, 100, 250, 500 ng per well) from Hycult Biotech (source #2), (**g**) oxPAPC (25, 50, 75, 100, 150 ng per well) and 150 ng per well oxPAPC in the absence of LPS from Hycult Biotech spontaneous oxidation (source #3), and (**h**) oxPAPC (25, 50, 75, 100, 150 ng per well) and 150 ng per well oxPAPC in the absence of LPS from Avanti Polar Lipids (source #4) as indicated. LDH release after 2h was determined as above. **i,** *Casp11*$^{-/-}$ BMDMs were primed with Pam3CSK4 (1 μg mL^{-1}) for 6h and then transfected with LPS (50 ng per well) and oxPAPC or PAPC (100 ng per well) and LDH release determined as above. **j,** BMDMs were mock transfected, transfected with biotinylated LPS (750 ng per 0.5 x 10^6 cells) or transfected with biotinylated LPS (750 ng per 0.5 x 10^6 cells) and oxPAPC (1.5 μg

per 0.5 x 10^6 cells) for 30 min and analyzed for intracellular LPS by flow cytometry. **k, l,** WT BMDMs were primed with (**k**) poly(I:C) (1 μg mL^{-1}) or (**l**) Pam3CSK4 (1 μg mL^{-1}) for 6h and then electroporated with LPS (50 ng per well) and oxPAPC or PAPC (100 ng per well) and LDH release determined as above. Data are representative of at least three independent experiments of at least triplicate samples. Error bars indicate ± s.d.; * P<0.05 by two-tailed unpaired *t*-test

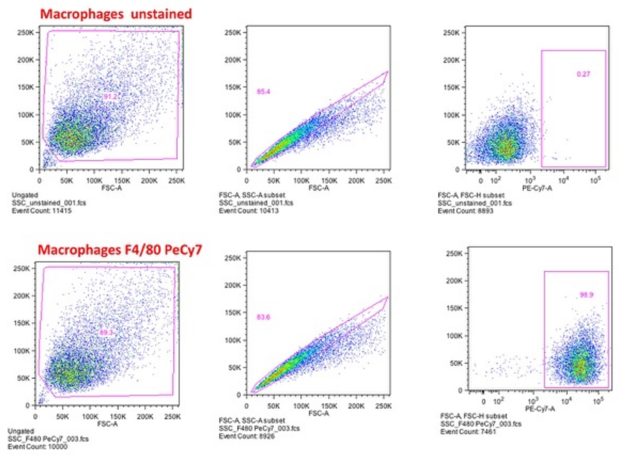

Supplementary Figure 2 | Gating Strategy for Supplementary Figure 1a

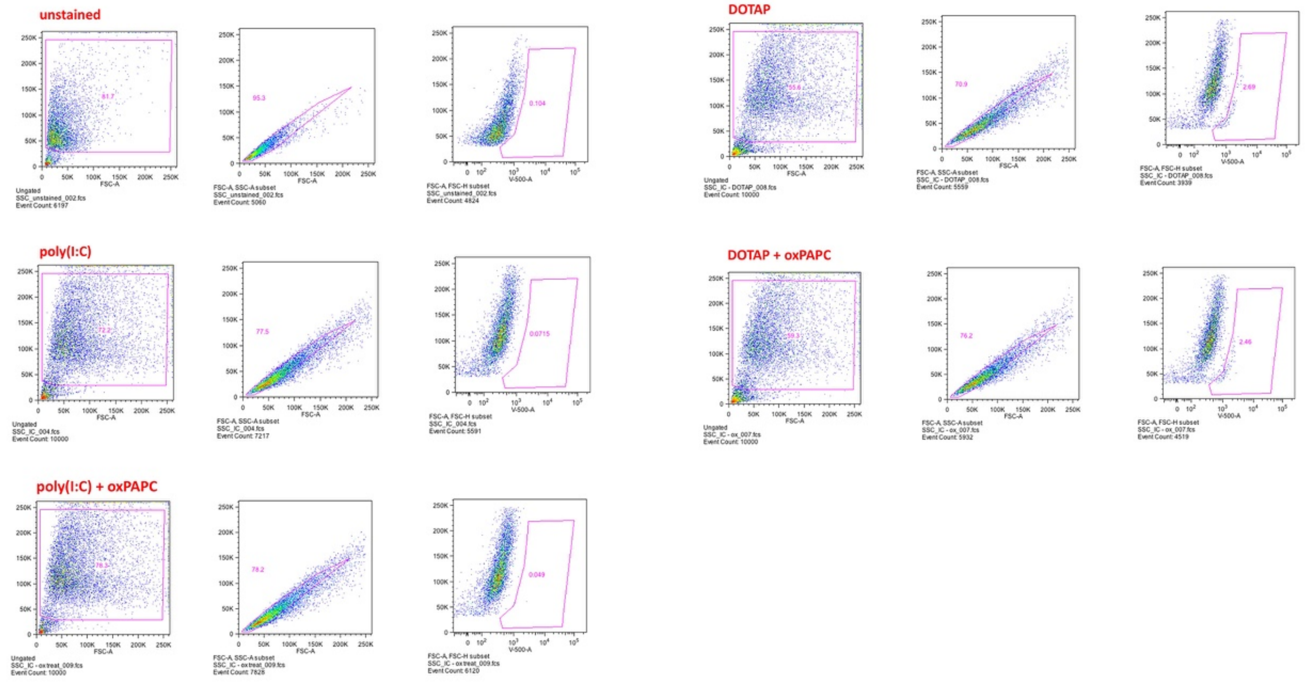

Supplementary Figure 3 | Gating Strategy for Supplementary Figure 1c

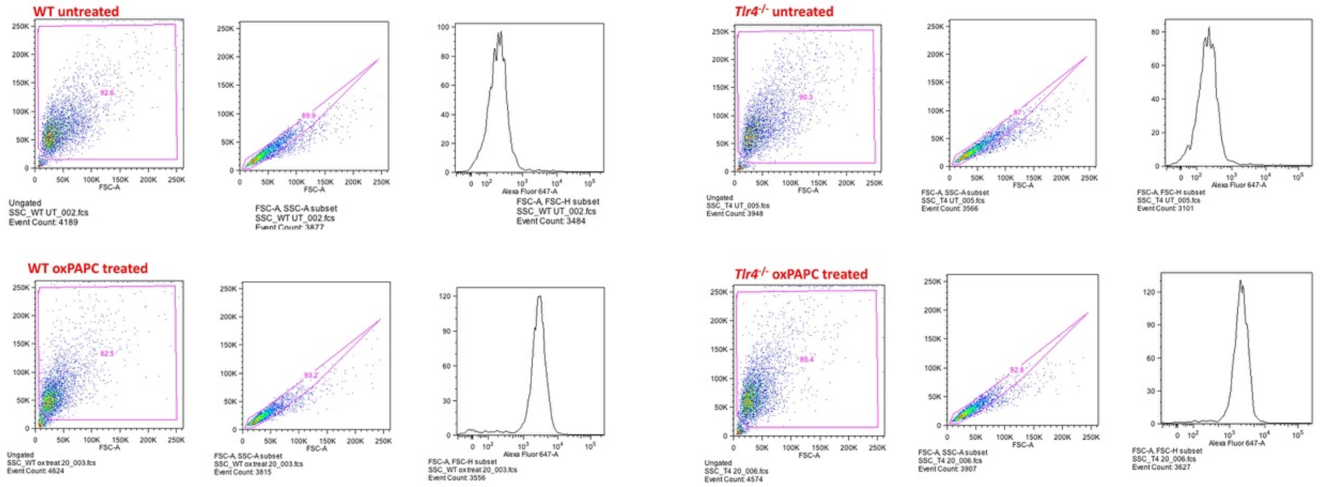

Supplementary Figure 4 | Gating Strategy for Supplementary Figure 1e

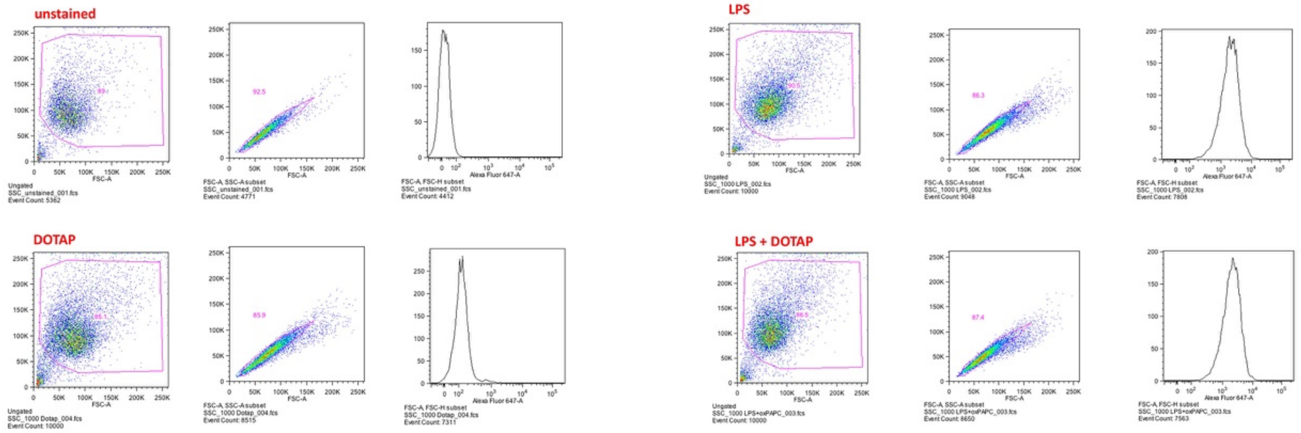

Supplementary Figure 5 | Gating Strategy for Supplementary Figure 1j

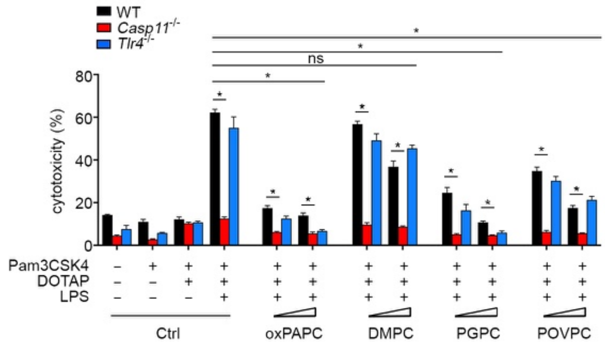

Supplementary Figure 6 | Phospholipids derived from PAPC peroxidation inhibit cytosolic LPS-induced pyroptosis WT, *Casp11*$^{-/-}$ and *Tlr4*$^{-/-}$ BMDMs were primed with Pam3CSK4 (1 μg ml^{-1}) for 6h and then transfected with LPS (50 ng per well) and either co-transfected with oxPAPC, DMPC, PGPC and POVPC (50 and 100 ng per well) and LDH release after 2h was determined and presented as % cytotoxicity compared to maximum LDH release. Data are representative of at least three independent experiments of at least triplicate samples. Error bars indicate ± s.d.; * $P<0.05$ by two-tailed unpaired *t*-test.

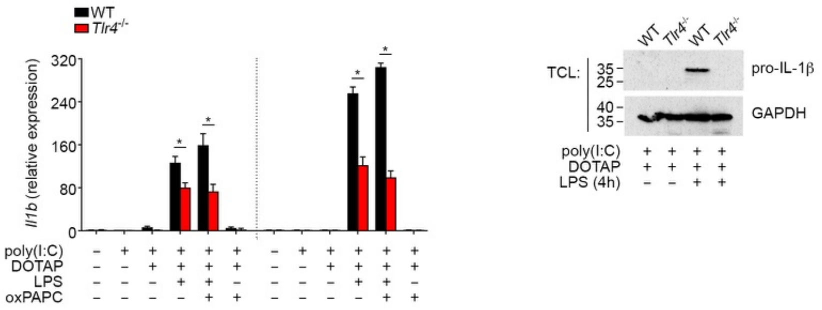

Supplementary Figure 7 | TLR4 is required for *Il1b* transcription following LPS transfection in macrophages. WT and *Tlr4*$^{-/-}$ BMDMs were left untreated, primed with poly(I:C) (1 μg mL^{-1}) for 6h and then mock transfected or transfected with LPS (50 ng per well) or LPS (50 ng per well) and co-transfected with oxPAPC (100 ng per well) and *Il1b* transcription was determined by qPCR after 1h and 2h or immunoblot after 4h. A molecular weight marker (kDa) is indicated. Data are representative of at least three independent experiments of at least triplicate samples. Error bars indicate ± s.d.; * P<0.05 by two-tailed unpaired *t*-test.

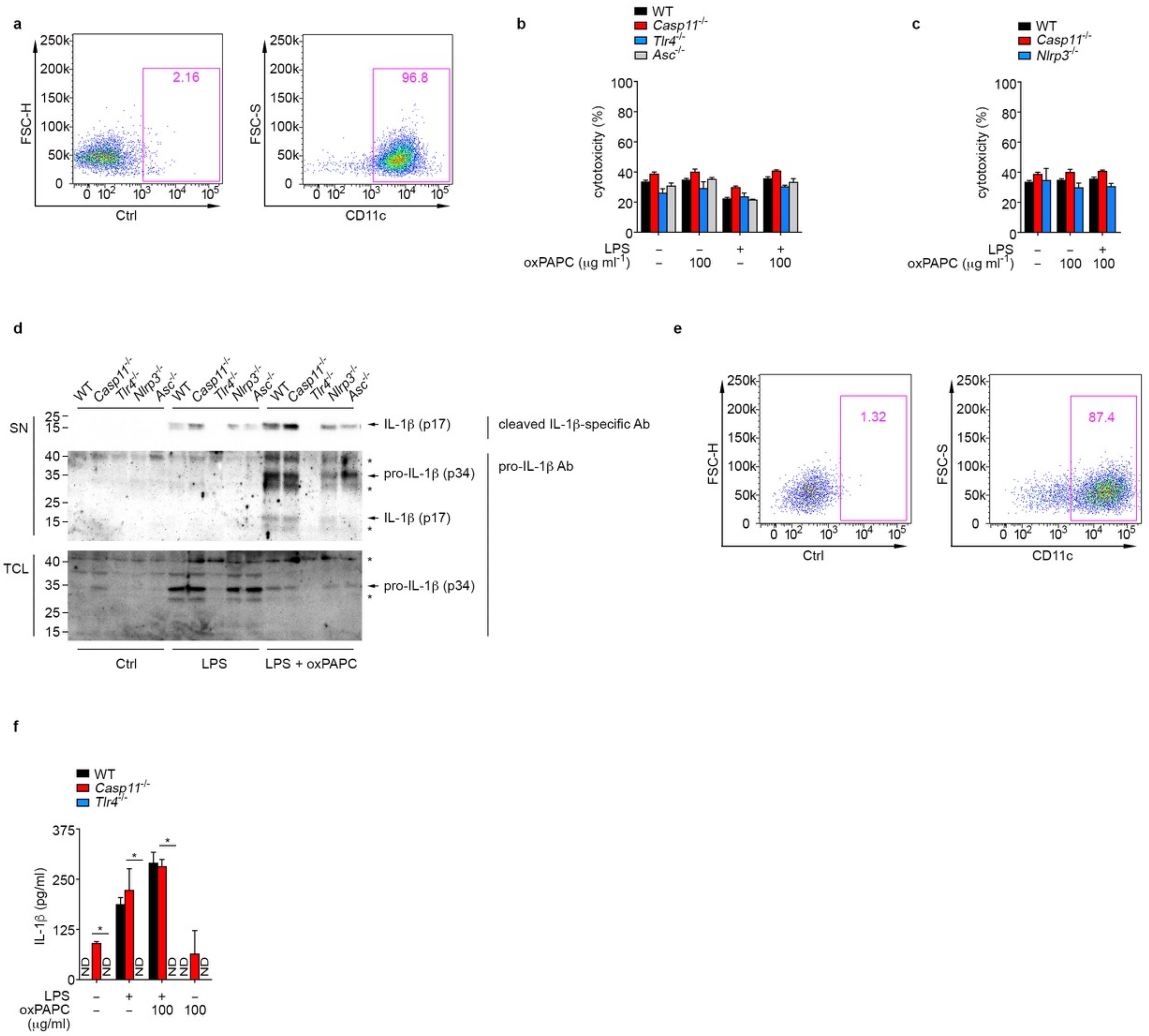

Supplementary Figure 8 | oxPAPC-induced IL-1β release in GM-DCs requires TLR4, but not Caspase-11. a, FLT3L/GM-CSF BMDCs were analyzed for CD11c expression by flow cytometry, indicating positive cells (%). **b, c,** BMDCs of the indicated genotypes were treated with LPS (1 μg ml^{-1}) for 3h and then treated with oxPAPC (100 μg mL^{-1}) or only oxPAPC (100 μg mL^{-1}) and culture supernatants were analyzed for LDH release 18h after LPS treatment and presented as % cytotoxicity compared to maximum LDH release. **d,** BMDCs of the indicated genotypes were treated as above and culture supernatants (SN) and total cell lysates (TCL) analyzed by immunoblot for pro-IL-1β and mature IL-1β using either an antibody specific for mature, cleaved IL-1β or pro-IL-1β**.** * denotes a cross-reactive protein. A molecular weight marker (kDa) is indicated. **e,** GM-CSF BMDCs were

analyzed for CD11c expression by flow cytometry, indicating positive cells (%). **f,** WT, *Casp11*$^{-/-}$ and *Tlr4*$^{-/-}$ BMDCs were treated with LPS (1 µg ml^{-1}) for 3h and then treated with oxPAPC (100 µg mL^{-1}) or only oxPAPC (100 µg mL^{-1}) and culture supernatants analyzed for secreted IL-1β by ELISA 18h after LPS treatment. Data are representative of at least three independent experiments of at least triplicate samples. Error bars indicate ± s.d.; * $P<0.05$ by two-tailed unpaired *t*-test.

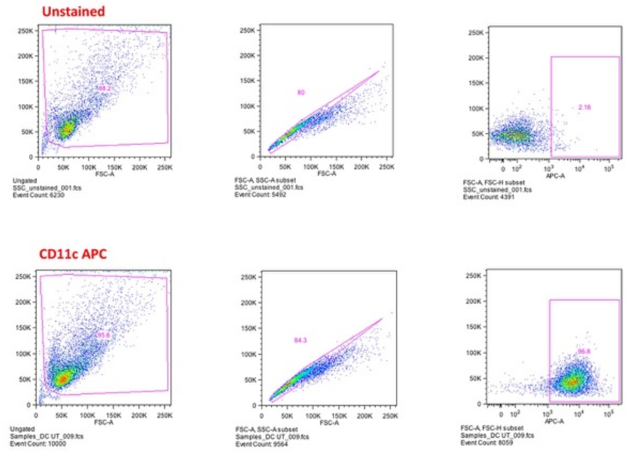

Supplementary Figure 9 | Gating Strategy for Supplementary Figure 8a

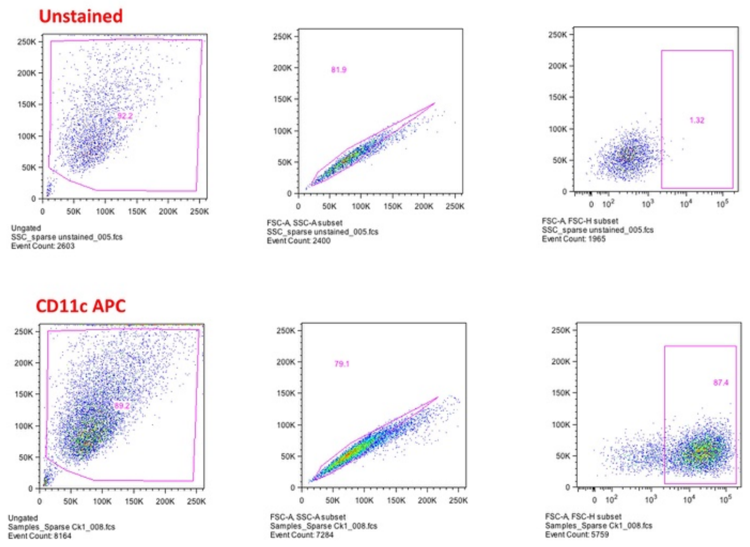

Supplementary Figure 10 ∣ Gating Strategy for Supplementary Figure 8e

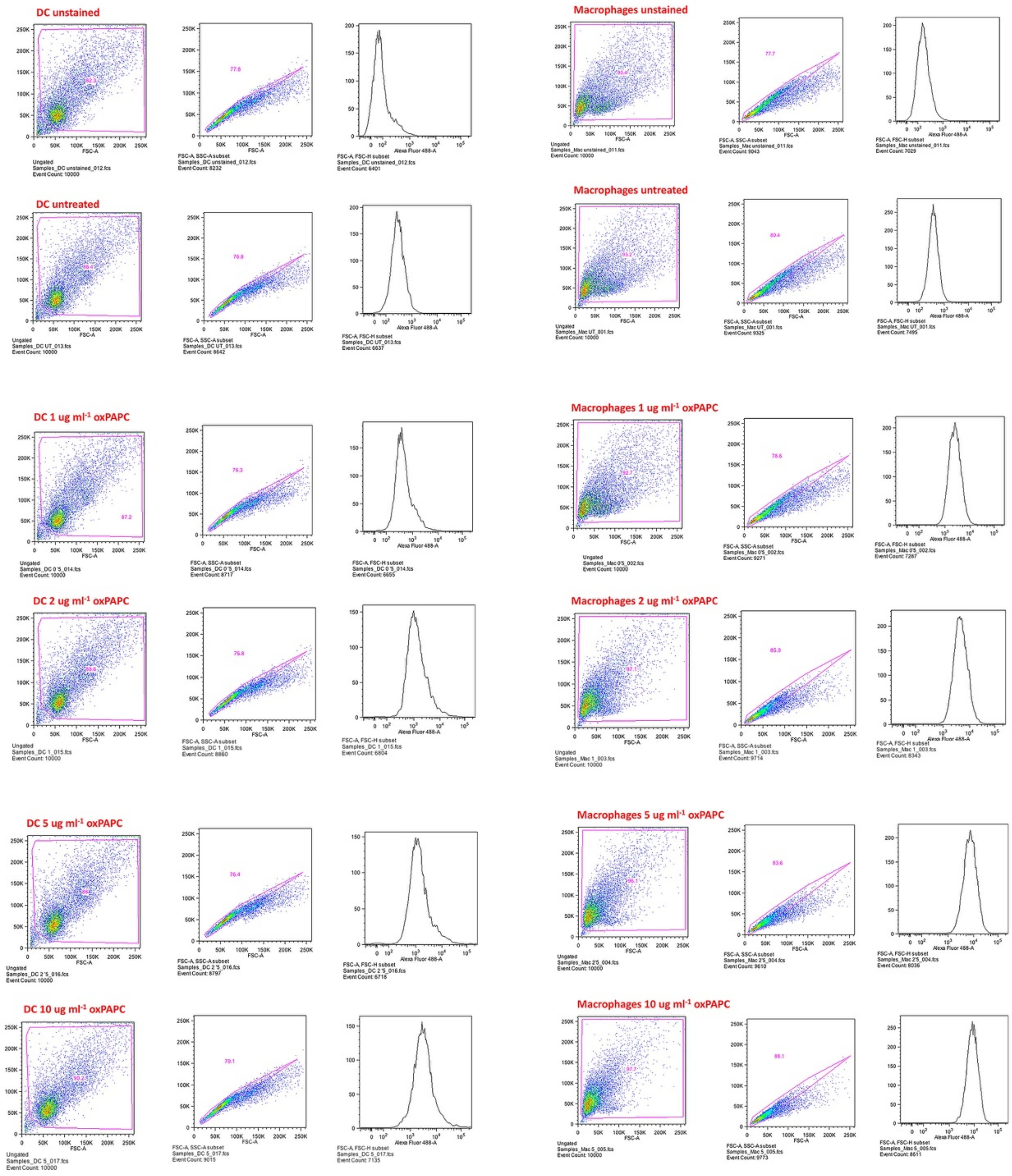

Supplementary Figure 11 | Gating Strategy for Figure 3d

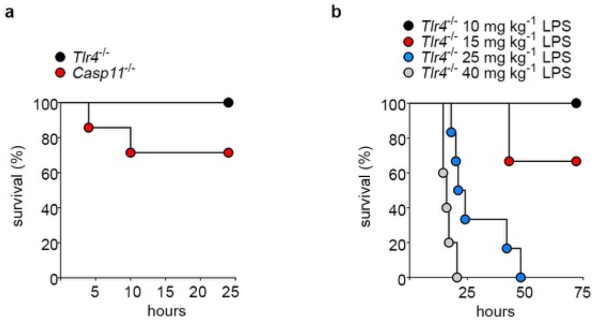

Supplementary Figure 12 | TLR4 contributes to LPS-induced lethality at low LPS concentrations a, *Tlr4*$^{-/-}$ and *Casp11*$^{-/-}$ mice (n=7) were i.p. injected with poly(I:C) (10 mg kg^{-1}) and 7h later i.p. injected with LPS (5 µg kg^{-1}) in Opti-MEM and survival was determined. **b,** *Tlr4*$^{-/-}$ mice (n=3-6) were primed with poly(I:C) as above and 7h later i.p. injected with LPS (10-40 mg kg^{-1}) in PBS and survival determined.

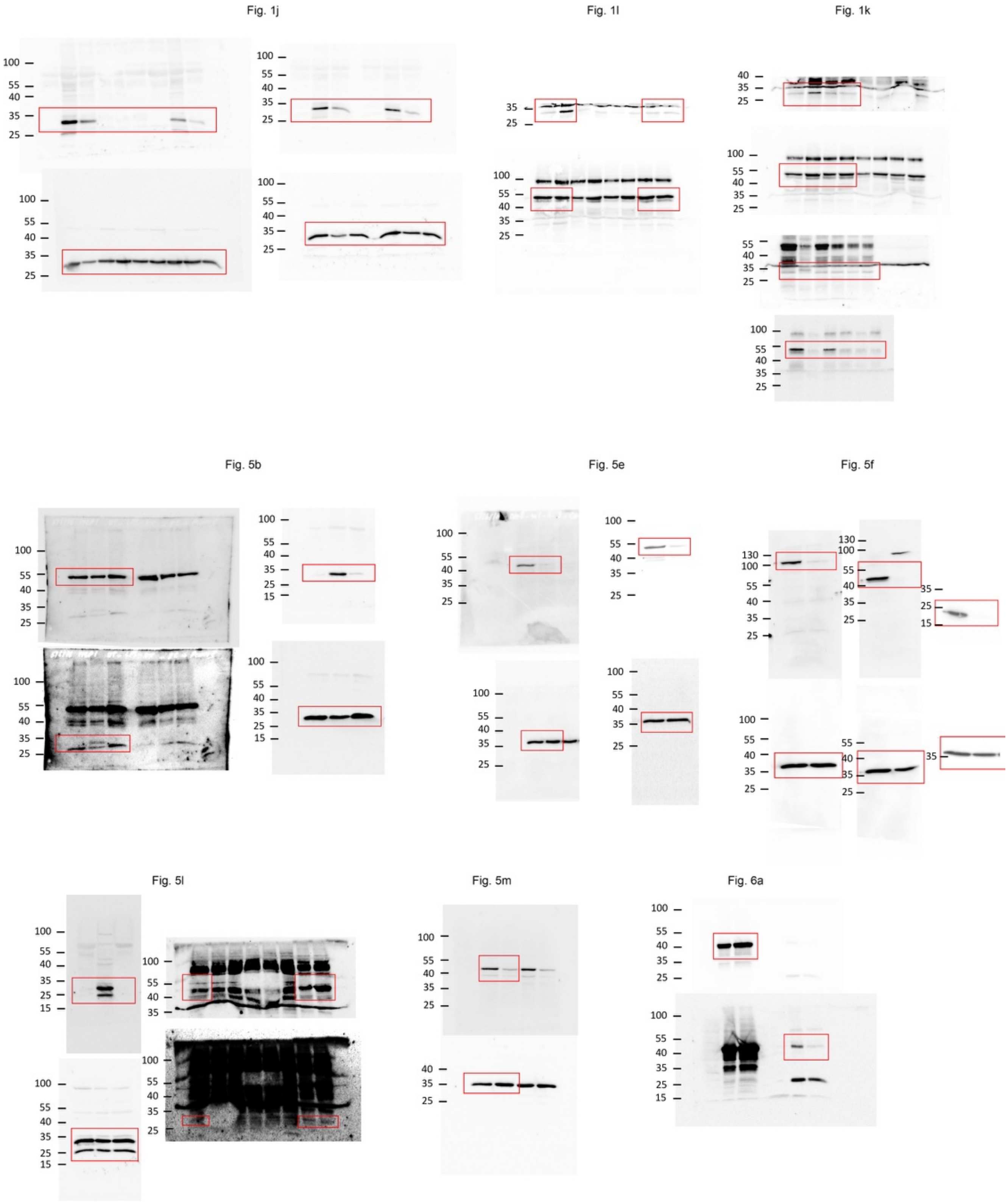

Supplementary Figure 13 | Uncropped Figures 1j, k, l; 5b, e, f, l, m; 6a. A molecular weight standard (kDa) is marked on the left.

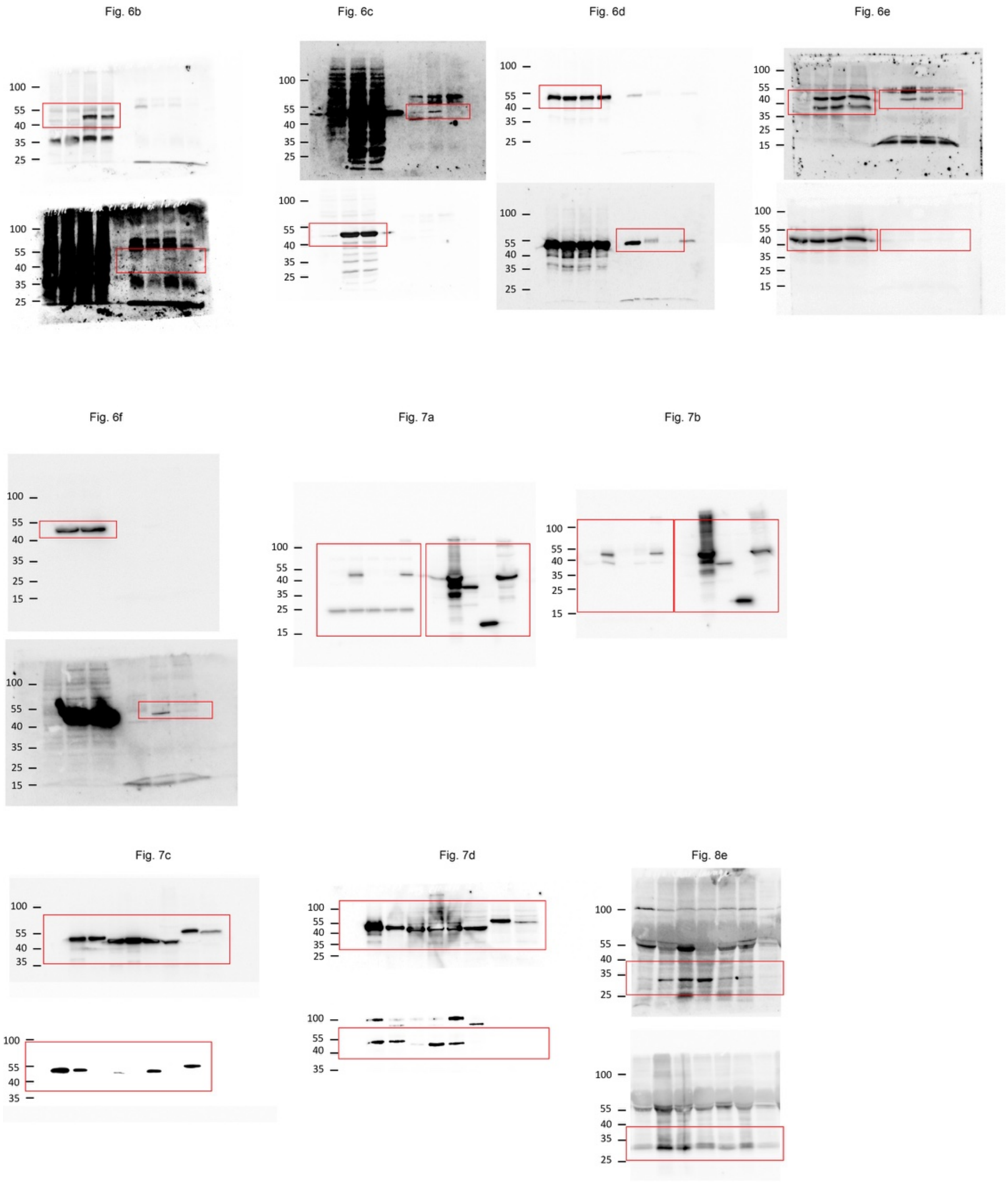

Supplementary Figure 14 | Uncropped Figures 6b-f; 7a-d; 8e. A molecular weight standard (kDa) is marked on the left.

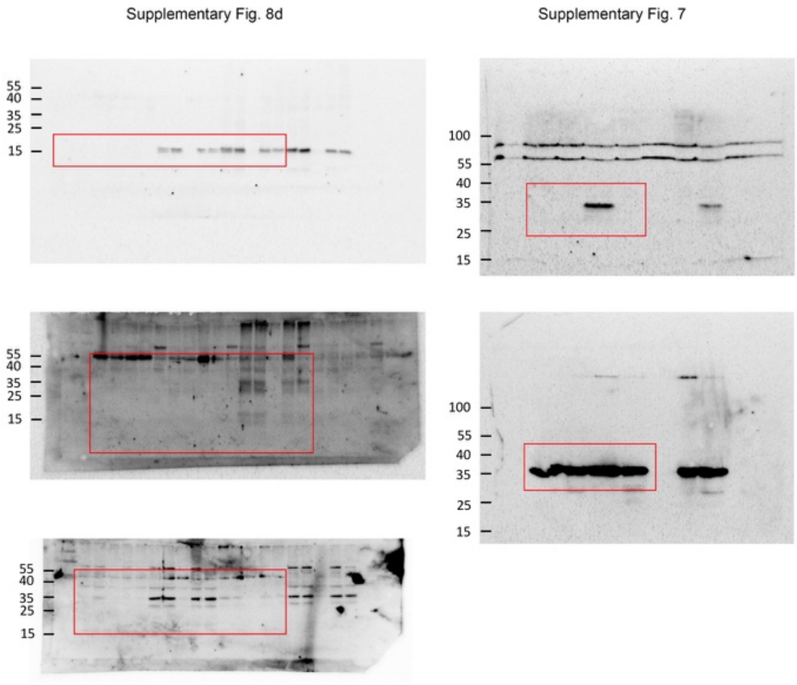

Supplementary Figure 15 | Uncropped Supplementary Figures 7 and 8d. A molecular weight standard (kDa) is marked on the left.
